# Nursing‐Integrated Photobiomodulation Therapy for Post‐Sternotomy Pain Management: A Retrospective Analysis of Acute Pain Alleviation and Opioid Sparing in a Single‐Center Cohort

**DOI:** 10.1155/prm/5561041

**Published:** 2026-08-30

**Authors:** Yan Wang, Yun Yu, Yubei Kang, Hua Jin, Zheyun Wang

**Affiliations:** ^1^ Nanjing Drum Tower Hospital, the Affiliated Hospital to Nanjing University Medical School, Nanjing, China, nju.edu.cn

**Keywords:** cardiac surgery, pain, photobiomodulation therapy, retrospective study

## Abstract

**Background:**

Postoperative pain is common among cardiac surgery patients, with some experiencing moderate to severe pain that persists for months. Photobiomodulation (PBM)—red light therapy, as a physical therapy, has been validated for promoting wound healing and alleviating neuropathic pain. This study aims to investigate the efficacy and safety of red light therapy in relieving acute postoperative pain in patients undergoing median sternotomy for cardiac surgery.

**Methods:**

This single‐center retrospective cohort study included 264 patients who underwent cardiac surgery at Nanjing Drum Tower Hospital between January 2024 and February 2025. Among them, 184 patients were used as the control group, and the patients in the control group did not receive red light therapy during their stay in the CICU, while 80 patients in the observation group received PBM within 2 h after entering the CICU on the day of surgery. The incidence of acute postoperative pain, critical‐care pain observation tool (CPOT) scores, morphine milligram equivalents per kilogram (MME/kg), and wound healing outcomes were compared between the two groups.

**Results:**

The incidence of acute pain was significantly lower in the observation group than in the control group (38.8% vs 57.1%, *p* < 0.05). Additionally, CPOT scores and MME/kg were significantly reduced in the observation group (*p* < 0.05). No significant differences were observed in postoperative drainage output or the incidence of poor wound healing between the two groups (*p* > 0.05).

**Conclusion:**

In this retrospective cohort, early application of red light therapy was associated with alleviated acute postoperative pain and reduced opioid consumption, with no observed increase in wound‐related complications. These findings suggest that PBM may serve as a safe and effective nonpharmacological adjunct for postoperative pain management in cardiac surgery patients.

## 1. Introduction

Approximately 50%–75% of patients experience varying degrees of pain after cardiac surgery, with some patients suffering from moderate to severe pain. Nearly 40% of patients continue to experience pain up to 6 months postsurgery [1–3]. The potential mechanism involves surgical trauma damaging nerve endings at the incision site, triggering local or systemic inflammatory responses, leading to peripheral and central sensitization [4]. Pain increases the risk of complications such as hospital‐acquired pneumonia and poor wound healing, delays postoperative recovery, and adds to the financial burden on patients [5]. Targeted reduction of inflammatory factors at the incision site may help alleviate postoperative pain in cardiac surgery patients, thereby improving their prognosis. Red light therapy, as a physical therapy, has been shown to reduce swelling, inflammation, and pain by improving tissue microcirculation and promoting leukocyte phagocytosis. It has been validated in studies on wound healing and neuropathic pain relief [6, 7]. This study aims to investigate whether early application of photobiomodulation (PBM) can effectively alleviate acute postoperative pain in cardiac surgery patients and assess its safety.

## 2. Subjects and Methods

### 2.1. Study Hypotheses

The primary hypothesis was that early postoperative application of red light therapy would reduce the incidence and severity of acute pain following median sternotomy compared to delayed therapy. Secondary hypotheses were that early red light therapy would reduce opioid consumption and would not increase wound‐related complications. In this study, “early therapy” was defined as the initiation of red light therapy on the day of surgery upon the patient’s return to the cardiac surgery intensive care unit (CICU). Conversely, “delayed therapy” referred to the initiation of red light therapy only after the control group patients were transferred to the general ward.

### 2.2. Study Subjects

This retrospective study was conducted in accordance with the ethical policies of Nanjing Drum Tower Hospital (Ethical Approval No. 2025–0095‐02), and patient informed consent was waived. The study included 264 consecutive patients who underwent elective cardiac surgery at Nanjing Drum Tower Hospital between January 2024 and February 2025. All patients received a standardized balanced general anesthesia protocol, including inhaled anesthetics, propofol, muscle relaxants, and remifentanil infusion. No additional long‐acting local anesthetics or regional nerve blocks were administered in either group. Due to the retrospective nature of the study, the precise intraoperative consumption of opioids could not be reliably ascertained. All patients were transferred to the CICU postoperatively. Among them, 184 patients admitted between January and June 2024 did not receive red light therapy during their CICU stay and served as the control group. Eighty patients admitted between July 2024 and February 2025 received red light therapy during their CICU stay and served as the observation group.

Inclusion criteria:

1. Patients undergoing elective cardiac surgery under general anesthesia.
2. Aged between 18 and 79 years.
3. Surgical approach via median sternotomy.
4. Mechanical ventilation duration ≤ 12 h.

Exclusion criteria:

1. History of malignant tumors.
2. Sensory dysfunction or psychiatric disorders.
3. Body mass index (BMI) < 18 kg/m^2^.
4. Previous thoracotomy.
5. Spinal cord injury.
6. Long‐term use of analgesic medications.
7. Reopening of the chest on the day of surgery (i.e., redo sternotomy due to active bleeding, cardiac tamponade, etc.).
8. Failure to wake up within 6 h postoperatively.
9. Patients requiring opioids for nonincisional pain.
10. Patients in the observation group who did not receive red light therapy until after the onset of acute postoperative pain.

Perioperative anesthetic and analgesic protocols were standardized at our institution. All patients received a balanced general anesthesia comprising inhaled anesthetics (sevoflurane), intravenous propofol, dexmedetomidine, fentanyl analogs, and muscle relaxants, titrated by the attending anesthesiologist according to routine clinical practice.

Postoperatively, fentanyl was used as the first‐line rescue analgesic for acute pain at our center. This practice diverges from some guideline recommendations that suggest routine NSAIDs as part of multimodal analgesia; however, we generally avoided NSAIDs in the early postoperative period for this cardiac surgery population, as their use at this stage has been associated with increased risks of bleeding and renal impairment—complications that could be life‐threatening in these patients. While fentanyl is effective for acute pain control, its well‐known side effects, including dependence and gastrointestinal discomfort, underscore the clinical need for nonpharmacological analgesic adjuncts such as PBM.

For background analgesia, a continuous intravenous infusion pump (without patient‐controlled bolus function) was used in all patients. According to the institutional routine protocol, the pump was prepared as 0.6 mg/kg of dezocine plus 16 mg of ondansetron diluted in 100 mL of normal saline and infused at a fixed rate of 2 mL/h. This weight‐adjusted, fixed‐rate regimen was administered identically to all patients in both groups as part of standard care.

With regard to adjunctive medications: Magnesium sulfate was routinely administered to all patients as part of the standard intravenous electrolyte supplementation protocol, rather than as a specific analgesic adjuvant. Dexamethasone was not used for routine analgesia; it was reserved solely for antipyretic purposes when clinically indicated (temperature > 38.5°C). Acetaminophen was not employed as a routine analgesic in the multimodal regimen; it was occasionally prescribed for antipyretic purposes when temperature exceeded 38.5°C. However, even when prescribed, actual administration was rarely executed, as the vast majority of patients had defervesced spontaneously by the time the medication arrived—a consequence of frequent pharmacy delivery delays. Given its sporadic and nonsystematic use, acetaminophen was excluded from the core analgesic analysis to avoid exposure misclassification bias.

Aspirin was routinely prescribed for patients undergoing coronary artery bypass grafting (CABG) as antiplatelet therapy, rather than for analgesic purposes. It is important to note that the anesthesia team for cardiac surgeries at our center consists of a fixed group of specially trained anesthesiologists who follow this unified perioperative protocol. All the above regimens were applied identically to patients in both the control and observation groups.

### 2.3. Research Methods

#### 2.3.1. Control Group Intervention

After surgery, patients were transferred to the CICU, where they received standardized postoperative care, including the use of a continuous infusion pump. Drainage tubes were properly secured to avoid displacement or kinking. If patients experienced acute pain, opioids were administered as prescribed. Patients in the control group received red light therapy only after transfer to the general ward, not during their CICU stay. The red light therapy was administered using a Carnation‐10 red light therapy device, with a wavelength of 640 ± 10 nm and a power density of ≥ 100 mW/cm^2^ at the center of the light output. The device was positioned 10–15 cm from the wound, with each session lasting 15 min, twice daily. Patients were provided with protective goggles during the therapy.

#### 2.3.2. Observation Group Intervention

In addition to the control group’s protocol, patients in the observation group began receiving red light therapy on the surgical incision on the day of their return to the CICU. The therapy was administered for 15 min per session, twice daily. Patients in the observation group received the first session of red light therapy within 2 h after returning to the CICU. Patients were transferred out of the CICU upon meeting standardized criteria: (1) hemodynamic stability without vasopressor support; (2) spontaneous breathing with a respiratory rate of 10–25 breaths per minute and oxygen saturation (SpO_2_) ≥ 94% on room air or with supplemental oxygen; and (3) a critical‐care pain observation tool (CPOT) score ≤ 1. The discharge decision was clinical parameter‐dependent and strictly adhered to these objective criteria, rather than being fixed to a specific postoperative day.

### 2.4. Evaluation Indicators

The study compared the following outcomes between the two groups: (1) incidence of acute postoperative pain; (2) time to first acute pain episode (T1), defined as the duration from the end of surgery to the patient’s first self‐report of incisional pain; (3) morphine milligram equivalents per kilogram (MME/kg), calculated based on rescue opioid boluses (primarily fentanyl) administered for breakthrough pain, excluding the fixed background infusion of dezocine (as the latter was delivered at a constant rate to all patients and did not influence intergroup comparisons); (4) CPOT scores; (5) postoperative drainage output on Postoperative Day 0 (the day of surgery) and on Postoperative Day 1; and (6) incidence of poor wound healing. The CPOT score was assessed by trained nurses at the following time points: upon patient admission to the CICU, upon awakening, upon patient complaint of pain, and routinely every 6 h, until patient transfer out of the CICU. Poor wound healing was defined as cases requiring additional surgical debridement postoperatively due to wound infection, hematoma, dehiscence, or persistent exudate. This information was obtained by reviewing operative or debridement notes in the medical records. The removal of postoperative drainage tubes for all patients followed a uniform protocol: Tubes were removed when drainage was less than 50 mL per day for two consecutive days, which typically occurred around 2 weeks postoperatively.

### 2.5. Data Collection and Quality Control

Data were collected by a professionally trained research team to ensure accuracy and consistency. Patient data were retrospectively collected from electronic nursing information systems and medical records. All data were managed electronically, with encrypted storage and backup to ensure data security. The study strictly adhered to the research protocol approved by the ethics committee to ensure the reliability of data collection and study results.

### 2.6. Statistical Analysis

Sample size was validated using G^∗^Power 3.1. Statistical analysis was performed using SPSS 27.0 software. Continuous data were expressed as x̄ ± *s* or median, and comparisons between groups were performed using *t* tests or nonparametric tests. Categorical data were expressed as frequencies and percentages, and comparisons were made using Fisher’s exact test or the chi‐square test. A *p* value of < 0.05 was considered statistically significant. The correlation of categorical variables was analyzed using binary logistic regression, with the model using the enter method.

## 3. Results

### 3.1. Sample Size Validation

The study included 184 patients in the control group and 80 in the observation group. The primary outcome was the occurrence of acute incisional pain postoperatively. To ensure sufficient statistical power, G^∗^Power 3.1 was used to validate the sample size. Based on previous literature [8], an effect size of 0.3 (medium effect) was assumed, with a significance level of 0.05 (two‐tailed test). With a sample size of 264, the calculated power was 0.90 (> 0.8), indicating that the sample size was adequate.

### 3.2. Comparison of Baseline Characteristics

The two groups showed differences in alcohol consumption history, smoking history, preoperative total protein levels, and time to awakening. No significant differences were observed in other baseline characteristics (*p* > 0.05), as shown in Table 1. Binary logistic regression analysis revealed no significant impact of alcohol consumption history, smoking history, preoperative total protein levels, or time to awakening on the incidence of acute postoperative pain, as shown in Table 2.

**TABLE 1:** Comparison of baseline characteristics between the two groups.

Variable	Control group (*n* = 184)	Observation group (*n* = 80)	Test statistic	*p* value
Sex				
Male (*n*, %)	107 (58.2)	51 (63.7)	0.727	0.394^1^
Age [years, *M* (P25, P75)]	61 (53,70)	60 (55,67)	−0.208	0.835^2^
No history of smoking (*n*, %)	141 (76.6)	45 (56.3)	11.126	< 0.001^1^
No history of alcohol consumption (*n*, %)	163 (88.6)	58 (72.5)	10.583	0.001^1^
No history of diabetes (*n*, %)	155 (84.2)	64 (80.0)	0.709	0.400^1^
BMI [kg/m^2^, M (P25,P75)]	24.13 (21.72,26.35)	24 (21.82,26,37)	−0.053	0.958^2^
Preoperative normalized international ratio [M (P25,P75)]	0.99 (0.94,1.04)	0.97 (0.93,1.04)	−1.084	0.279^2^
Preoperative hemoglobin [g/L, M (P25,P75)]	133.5 (124,145)	135 (123.25,146)	−0.165	0.869^2^
Preoperative total protein^4^ (g/*L*, x̄ ± *s*)	65.74 ± 5.36	62.78 ± 5.89	3.990	< 0.001^3^
Length of surgery [h, *M* (P25, P75)]	4.50 (3.90,5.40)	4.40 (3.83,5.20)	−0.504	0.614^2^
Surgical method (*n*, %)			1.517	0.678^1^
Cardiac valve surgery	81 (44)	33 (41.3)		
Coronary artery bypass grafting	49 (26.6)	26 (32.5)		
Cardiac macrovascular surgery	11 (6.0)	6 (7.5)		
Hybridization surgery	43 (23.4)	15 (18.8)		
Number of drains (*n*, %)			3.741	0.291^1^
1	11 (6.0)	1 (1.3)		
2	140 (76.1)	60 (75.0)		
3	29 (15.8)	17 (21.3)		
4	4 (2.2)	2 (2.5)		
Mechanical ventilation time [h, M (P25,P75)]	7 (5,8)	6 (5,9)	−0.630	0.529^2^
Awakening time^5^ [h, *M* (P25, P75)]	2 (2,3)	2 (1,3)	−2.115	0.034^2^

^1^X^2^ test.

^2^Mann–Whitney *U* test.

^3^ *T* test.

^4^Total protein is composed of albumin and globulin. It is one of the common indicators for screening liver function and nutritional status.

^5^Awakening time was defined as the duration from the end of surgery to the patient’s first response to verbal commands.

**TABLE 2:** Binary logistic regression analysis of the incidence of acute postoperative pain after cardiac surgery.

Variable	Regression coefficient	Standard error	Chi‐square value	OR	95% CI for OR	*p* value
Group	0.824	0.313	6.924	2.28	1.234∼4.214	0.009
Age	−0.029	0.014	4.543	0.971	0.946∼0.998	0.033
BMI	0.046	0.042	1.209	1.048	0.964∼1.138	0.272
Length of the operation	−0.091	0.129	0.505	0.913	0.709∼1.175	0.477
Awakening time	0.013	0.135	0.01	1.014	0.778∼1.320	0.92
Mechanical ventilation time	−0.114	0.073	2.426	0.893	0.774∼1.030	0.119
Preoperative hemoglobin	0.009	0.01	0.879	1.009	0.990∼1.029	0.349
Preoperative total protein	−0.032	0.026	1.604	0.968	0.921∼1.018	0.205
Preoperative INR	−0.462	0.729	0.402	0.63	0.151∼2.629	0.526
Surgical method			4.116			0.249
Cardiac valve surgery	−0.018	0.359	0.003	0.982	0.486∼1.984	0.959
Coronary artery bypass grafting	0.738	0.445	2.757	2.092	0.875∼5.000	0.097
Cardiac macrovascular surgery	0.129	0.591	0.047	1.137	0.357∼3.625	0.828
Number of drains			5.664			0.129
1	2.586	1.433	3.258	13.281	0.801∼220.218	0.071
2	0.117	0.88	0.018	1.125	0.200∼6.311	0.894
3	0.497	0.928	0.286	1.643	0.266∼10.139	0.593
Sex	−0.308	0.387	0.633	0.735	0.345∼1.569	0.426
No history of diabetes	−0.043	0.42	0.01	0.958	0.421∼2.180	0.918
No history of smoking	0.313	0.381	0.675	1.368	0.648∼2.888	0.411
No history of alcohol consumption	0.012	0.459	0.001	1.012	0.412∼2.487	0.979
constant	2.089	2.762	0.572	8.081		0.449

*Note:* The length of CICU stay showed no significant difference between the two groups (control vs. observation: 2 (2,3) days vs. 2 (2,3) days, *Z* = −0.607, *p* = 0.544). Patients in the observation group received an average of 4 sessions of red light therapy during their CICU stay (twice daily on Postoperative Days 0 and 1).

### 3.3. Comparison of Postoperative Outcomes

The incidence of acute pain and CPOT scores were significantly lower in the observation group compared to the control group (*p* < 0.05). There was no statistically significant difference between the two groups in terms of drainage output on the day of surgery, on Postoperative Day 1, or in the incidence of poor wound healing (*p* > 0.05), as presented in Table 3. Among patients who experienced acute pain, there was no significant difference in the time to pain onset (*p* > 0.05), but the MME/kg was significantly lower in the observation group (*p* < 0.05), as shown in Table 4.

**TABLE 3:** Comparison of pain relief effects and safety in the two groups.

Variable	Control group (*n* = 184)	Observation group (*n* = 80)	Test statistic	*p* value
Acute pain (*n*, %)	105 (57.1)	31 (38.8)	7.488	0.006^1^
CPOT score distribution (*n*, %)			9.999	0.040^1^
0 points	79 (42.9)	49 (61.3)		
1 point	19 (10.3)	3 (3.8)		
2 points	50 (27.2)	20 (25.0)		
3 points	29 (15.8)	6 (7.5)		
≥ 4 points	7 (3.8)	2 (2.5)		
Drainage output, Postoperative Day 0 [ml, M (P25, P75)]	355 (240, 497.5)	300 (220, 410)	−1.884	0.060^3^
Drainage output, Postoperative Day 1 [ml, M (P25, P75)]	250 (200, 320)	230 (172.5, 317.5)	−1.311	0.190^3^
Poor wound healing (*n*, %)	11 (6.0)	1 (1.3)	2.873	0.114^2^

^1^ *X* ^2^ test.

^2^Fisher’s exact test.

^3^Mann–Whitney *U* test.

**TABLE 4:** Comparison of pain‐related indicators in the two groups of patients with acute pain attacks.

Variable	Control group (*n* = 105)	Observation group (*n* = 31)	*Z* value	*p* value
T1[h,M (P25,P75)]	11 (7, 24)	13 (6, 25)	−0.620	0.535
MME/kg [M (P25,P75)]	2.33 (1.32, 3.20)	1.42 (0.76, 2.78)	−2.311	0.021

## 4. Discussion

### 4.1. Early Red Light Therapy Reduces the Incidence and Severity of Acute Postoperative Pain in Cardiac Surgery Patients

Although there were differences in baseline characteristics such as smoking history, alcohol consumption history, preoperative total protein levels, and time to awakening between the two groups (Table 1), binary logistic regression analysis indicated that these factors did not have a significant impact on the incidence of acute postoperative pain (Table 2). Therefore, the observed differences in pain outcomes between the groups can be primarily attributed to the red light therapy intervention. As shown in Table 3, the incidence of acute postoperative pain and CPOT scores were significantly lower in the observation group compared to the control group (*p* < 0.05), which is attributed to the early application of red light therapy in the observation group. Cardiac surgery induces inflammatory responses that stimulate nociceptors around the incision, releasing pain‐inducing substances and causing acute pain [9–11]. Red light therapy, with its noninvasive cold light at a wavelength of 600–700 nm, penetrates dressings and acts on subcutaneous tissues, reducing the release of inflammatory and oxidative factors, thereby alleviating pain [12, 13]. Consistent with Bjornnes et al. [14], the median time to acute pain onset was 11–13 h postoperatively. Patients in the observation group received red light therapy before the onset of acute pain, which suppressed inflammatory factors at the incision site, reduced hyperalgesia, and increased the pain threshold [15, 16]. This led to a reduction in the subjective perception of pain and a decrease in the incidence of acute pain. Additionally, the control group, which did not receive early red light therapy, exhibited increased pain sensitivity, resulting in an earlier median time to pain onset (T_1_). The findings suggest that early red light therapy is effective in alleviating acute postoperative pain in cardiac surgery patients, although further research is needed to determine the optimal timing and duration of the intervention.

### 4.2. Early Application of Red Light Therapy in Cardiac Surgery Is Safe

No significant differences were observed in postoperative drainage output or the incidence of poor wound healing between the two groups (*p* > 0.05). However, the median drainage output and incidence of poor wound healing were lower in the observation group, suggesting that red light therapy may promote wound healing and fluid absorption in cardiac surgery patients. Among patients who experienced acute pain, the MME/kg was significantly lower in the observation group (*p* < 0.05). This can be attributed to the higher overall pain levels in the control group, leading to increased opioid demand, which in turn exacerbated opioid‐induced hyperalgesia and dependence [16]. Red light therapy reduced pain sensitization in the observation group, further decreasing the need for opioids. Reducing opioid dependence not only lowers respiratory risks but also minimizes side effects such as weakness and drowsiness, which is particularly important for early postoperative recovery, especially in elderly patients. Compared with regional anesthesia techniques (e.g., parasternal nerve blocks), PBM offers distinct practical advantages in this setting. It is entirely noninvasive, carries no risk of pneumothorax, vascular injury, or local anesthetic toxicity and can be safely administered by trained nursing staff on the general ward. Crucially, unlike single‐injection nerve blocks which typically wane within 12–24 h, nurse‐integrated PBM could be delivered repeatedly on a daily basis throughout the early postoperative period, providing sustained analgesia that aligns well with the trajectory of acute incisional pain. Although red light therapy has been widely used for pain relief and wound healing, its application in cardiac surgery is relatively new, and there are limited data on its safety in this population. This study demonstrates that early application of red light therapy in cardiac surgery is safe. Given that cardiac surgery patients are routinely admitted to the CICU, where emergency measures are more readily available, further research on early red light therapy is warranted to obtain more comprehensive clinical data and optimize nonpharmacological pain management strategies in cardiac surgery.

### 4.3. Red Light Therapy Improves Patient Experience and Optimizes Cost Structure

Postoperative patients often experience multiple adverse events, including mechanical ventilation, weaning exercises, pain, and thirst [17]. Some patients may experience a cumulative effect, exacerbating physical discomfort. Compared to the control group, the observation group had lower overall CPOT scores and a delayed median time to acute pain onset (T_1_). From the perspective of the “peak‐end rule” [18], reducing pain intensity and delaying pain onset can lower the peak of adverse experiences, thereby improving postoperative recovery and patient satisfaction. Patients in the observation group who received early red light therapy experienced a significant reduction in the incidence and intensity of acute pain, leading to decreased opioid demand and dependence. This not only directly reduces the cost of analgesic medications but also indirectly lowers the costs associated with opioid‐related side effects such as constipation and hiccups. This shift reduces the proportion of clinical drug costs while increasing the proportion of technical service fees. Given the seasonal nature of cardiovascular disease [19, 20] and the influence of factors such as bed availability on hospital stay duration, this retrospective study did not include hospital stay length or costs in the statistical analysis. Future prospective studies should control for relevant variables to further analyze the impact of red light therapy on hospital stay duration and costs.

### 4.4. Strengths and Limitations

The main strengths of this study are its relatively large sample size and the analysis of real‐world clinical practice. However, as a single‐center retrospective study, it has several limitations. First, although we adjusted for measured baseline differences, we acknowledge the potential for residual confounding. Specifically, despite all patients receiving a standardized general anesthesia protocol with fentanyl analogs as the primary intraoperative opioids, the precise cumulative intraoperative fentanyl dosage was not reliably retrievable from the electronic records, as it was titrated individually by the attending anesthesiologist. Nevertheless, because the same standardized protocol and the same team of anesthesiologists were applied across both groups, any undocumented intraoperative variation is unlikely to have systematically biased the postoperative pain outcomes. Second, the treatment parameters (e.g., wavelength, power density, duration of red light therapy) were fixed, precluding the determination of an optimal treatment protocol. Furthermore, the nonrandomized, time‐segmented allocation of patients (control group from the first half of 2024 vs. observation group from the second half of 2024 onward) may introduce time‐related biases that could not be fully adjusted for, such as seasonal variations in patient acuity or unmeasured evolution in institutional pain management practices over the study period. Finally, this study lacks follow‐up data on patients′ long‐term pain outcomes, functional recovery, or quality of life, which should be explored in future prospective studies.

## 5. Conclusion

This retrospective study suggests that early postoperative red light therapy may be a safe and effective nonpharmacological intervention for alleviating acute pain and reducing opioid requirements in cardiac surgery patients undergoing median sternotomy. Prospective studies are warranted to determine optimal treatment parameters and validate its cost‐effectiveness.

## Author Contributions

**Yan Wang**: data curation (equal); formal analysis (equal); investigation (equal); project administration (equal); and writing–original draft (lead). **Yun Yu**: data curation (equal); formal analysis (equal); project administration (equal); and writing–original draft (equal). **Yubei Kang**: data curation (equal) and project administration (equal). **Hua Jin**: data curation (equal). **Zheyun Wang**: data curation (equal); formal analysis (equal); investigation (equal); project administration (equal); and writing–review and editing (equal).

## Funding

This study was supported by Nanjing Drum Tower Hospital Nursing Research Project (No. 2024‐B648).

## Ethics Statement

Ethical approval was obtained from the Ethics Committee of Nanjing Drum Tower Hospital (Approval No: 2025‐0095‐02). The requirement for informed consent was waived due to the retrospective nature of the study.

## Conflicts of Interest

The authors declare no conflicts of interest.

## Data Availability

The data that support the findings of this study are available on request from the corresponding author. The data are not publicly available due to privacy or ethical restrictions.
